# From Living in Saltwater to a Scarcity of Salt and Water, and Then an Overabundance of Salt—The Biological Roller Coaster to Which the Renin–Angiotensin System Has Had to Adapt: An Editorial

**DOI:** 10.3390/biomedicines11113004

**Published:** 2023-11-09

**Authors:** György L. Nádasy, András Balla, Mária Szekeres

**Affiliations:** 1 Department of Physiology, Faculty of Medicine, Semmelweis University, 37-47 Tűzoltó Street, 1094 Budapest, Hungary; nadasy.gyorgy@med.semmelweis-univ.hu (G.L.N.); balla.andras@med.semmelweis-univ.hu (A.B.); 2 Laboratory of Molecular Physiology, Eötvös Loránd Research Network, Research Centre for Natural Sciences, 2 Magyar Tudósok Körútja, 1117 Budapest, Hungary; 3 Department of Morphology and Physiology, Faculty of Health Sciences, Semmelweis University, 17 Vas Street, 1088 Budapest, Hungary

Angiotensin II (Ang II) is a hormone with much more complex actions than is typical for other agonists with heterotrimeric G protein-coupled receptors (GPCRs). Its principal receptor, angiotensin type 1 receptor (AT_1_R), is distributed in many cells of the body in diverse organs, controlling the water and salt balance, vascular contractility and blood pressure. The main functions are discussed below.

## 1. Pleiotropic Effects of Ang II

AT_1_Rs can attach promiscuously to G_q/11_, Gi/o and G_12/13_ intracellular signal proteins in various cells of the body. The activation of the mitogen-activated protein kinase (MAPK) cascade and the release of epithelial growth factor (EGF) and vascular endothelial growth factor (VEGF) lend a trophic character to the hormone [1,2,3]. In the blood vessel wall, it induces inflammatory cell transformation, chronic inflammation with cytokine release, white cell migration, fibrosis and apoptosis of cells. It can be considered an inflammatory transmitter with a strong resemblance to chemokines [4]. It is also a neural transmitter. In the brain stem, diencephalon and limbic system neural circuits controlling salt and water balance even behavior elements containing Ang II-releasing neurons [5,6]. These complex actions are very far from the original epithelial salt-conserving function of this archaic agonist. In the mammalian body, several functions intermingle inseparably in the diverse actions of Ang II. Corrections needed to restore the salt balance, volume balance, blood pressure, vascular mechanics, cellular longevity and drinking behavior in optimal cases may be parallel, but it could be that the adjustment of one important parameter induces disadvantageous changes in another. In the case of fast environmental changes, with no time for genetically fine-tuning the whole system, *Ang II will emerge as a pathological factor*. 

Three papers which have been published in this Special Issue deal with such pleiotropic functions of the renin–angiotensin system (RAS). In an excellent study by Miotto et al. [7] from the State University of Sao Paulo, Brazil, the aortic wall of perindopril-treated spontaneously hypertensive rats (SHR) was analyzed. Using the best available high throughput proteomic methods, they found that chronic treatment with the angiotensin converting enzyme (ACE) inhibitor perindopril in the artic wall altered 38 subcategories of cellular protein components, among them “supramolecular polymer”, “heterotrimeric G protein complex”, “actin cytoskeleton”, “supramolecular fiber”, “intermediate filament”, “membrane raft” and “oxidoreductase” complexes. The reduced aortic stiffness they observed seems to be connected to the elevated expression of the Ehd2 (EH domain-containing) protein, a component involved in the endothelial nitric oxide synthase (eNOS)–nitric oxide (NO) endothelial vasodilatory pathway. They succeeded in proving that the rigidity-reducing effect of chronic exercise has a different mechanism [7]. The extremely complex nature of the renin–angiotensin system (RAS) is shown by another paper in this Special Issue. Danilov et al. [8] found that the steric structure of the ACE protein is dependent not only on its amino acid sequence, but also on the presence of certain blood constituents [8]. Pathological connections of the RAS are far-reaching. Interspecies conservatism of the structure of ACE2, its receptor protein, made it possible for the SARS-CoV-2 virus to adapt from the original bat host to humans with surprising speed. A virus binding to the attacked cells will be followed by its internalization, together with the receptor. The elements of RAS will be involved in several subsequent pathological events. Such long post-COVID cardiovascular injuries have been reviewed by Cojocaru et al. in a recent Special Issue [9]. 

## 2. The Natural History of Water and Salt Balance

If we want to understand the role of Ang II as an important pathological factor, we have to explore how its problematic, divergent characteristics developed. The types of life occupying the disturbingly thin (12 miles) biosphere on the surface of the Earth are inherently attached to highly organized macromolecular interactions in watery solutions. Water, one of the most abundant “liquid stones” of the Earth’s crust, diluted salty substances from the solid rocks. Early forms of life adapted to that salty water, the salinity of which increased with time. Our planet can be considered extremely fortunate, having been able to keep water and maintain it in its fluid form on its surface for such a long time. This has ensured the continuous existence and development of life. With the rising of the continents, dry land appeared, forming new habitats for living creatures that they were eager to colonize. On continents, water needed for life processes could be ensured through the atmospheric water circulation: rain, molten snow and ice watered the soil and fed rivers and lakes. However, this water was only of very limited salt content. Sophisticated mechanisms developed to keep the water and salt balance of the organisms even under such conditions [10,11,12].

Marine invertebrates succumbed to the slowly elevating salinity of surrounding seawater, their extracellular osmolarity elevated in equilibrium with it. Their angiotensin II hormone controlled the extracellular fluid pressure and volume. In marine fish, however, like in all vertebrates, extracellular fluid osmolarity conserved the lower salinity of the ancient oceans. This was a mere third of present day’s ocean salinity values. Keeping extracellular sodium values constant simplified the genetic maintenance of the elaborate membrane ionic processes on which their sophisticated and, in the case of vertebrates, very successful neural and muscle functions depended. For marine vertebrates, the removal of salt from the body turned out to be the most important task. In fishes, it is performed due to the esophageal desalination of swallowed sea water and sodium excretion through the epithelial ionocytes of the gills [13]. The RAS controls extracellular fluid volume (thirst, determining salty sea-water swallowing), as well as the contraction of the newly acquired blood vessels that form a closed “intravascular” compartment lined with a smooth, continuous, low-friction endothelial sheath and, in case of larger vessels, additionally surrounded by a contractile smooth muscle media. Adjustments in blood volume and vascular and heart contractilities ensure proper intravascular blood flow and pressure. All these parameters are controlled (among others) by the renin–angiotensin system, through the action of the renin enzyme secreted into the blood by the mesonephros and of the peripheral ACE producing the active angiotensin II octapeptide. The kidneys of fishes are mesonephros, where nephrons do not have a Henle loop [14]. The mesonephros is present in the human embryo. It is fully developed at the 8th week and disappears at embryonal week 16, giving way to the metanephros [15]. Fish species living in freshwater rivers and lakes are confronted with just the opposite task: while water was still in abundancy (if a draught did not happen), there was a scarcity of salt. The lack of salt should be prevented by proper sodium reuptake processes in the kidneys and uptake from the low-sodium freshwater habitats. The very long shores of rivers, lakes, rivulets and swamps made it a successful strategy to live partially (amphibians) or permanently (reptiles, birds, mammals) on dry land where, however, both water and salt were scarce. From that point onward, ensuring both water and salt requirements of the body, and economizing them, became one of the most important tasks to ensure survival. Quadrupeds living on dry land for a substantial part of their life could only manage it through their sophisticated kidneys, the metanephros, with very effective sodium reuptake mechanisms in the Henle loop, distal and connecting tubules, as well as in the collecting duct, controlled both directly and indirectly (mineralocorticoid) through metanephric JGA renin and peripheral angiotensin II. The metanephros in the human fetus starts to develop at week 5 and remains thereafter in the final form of the kidney [15]. While renin-containing granules are distributed in the cells along the length of the afferent arteriole in fish mesonephros and early fetal metanephros, renin production is concentrated in the juxtaglomerular apparatus in adult mammals: granular cells of the afferent arteriole in the vicinity of the macula densa of the distal tubules store and release the renin controlled by (among other factors) the amount of salt in the distal tubules [16]. An effective osmoregulation ensures corresponding water conservation. 

## 3. The Natural History of the Elements of RAS

As we can see, the renin–angiotensin system plays an important role in maintaining the salt and water balance of very diverse animal species under very diverse conditions. It may be surprising that the components of the renin–angiotensin system are highly conserved proteins and peptides, and that their basic molecular structure has been maintained during their evolution. Vertebrate angiotensin peptides differ from each other in no more than 2–3 amino acid residues [17]. Functionable ACE is present in Gram-negative bacteria, annelid worms and molluscs [18,19]. Vertebrate development has been associated with the duplication of the ACE gene, where a more active form of the enzyme developed and the ancestral unduplicated form of the protein retained its role in testicular development (tACE) [20]. The angiotensin II stimulation of adrenocortical cells to produce mineralocorticoid appeared in early fish (shark) [21]. Renin production in the kidney, peripheral ACE, angiotensin I and II production and angiotensin receptors are present in all vertebrate classes (with the potential exemption of some lower fishes) [17]. Angiotensin II contracts fish blood vessels either directly or through catecholamine release and elevates blood pressure, which has a central dipsogenic effect [16]. The angiotensin receptors (AT_1_R and AT_2_R) have substantial homology with chemokine receptors, apelin receptors and even opioid receptors, which can explain their involvement in inflammatory, cell differentiation and neurotransmitter processes [22]. The ancestry of RAS in sea life can be judged from the fact that extracts of several marine microbial fungal strains have been found to effectively inhibit the mammalian AT_1_ receptor (also ET_A_ and ET_B_ receptors). At the same time, this observation demonstrates what pharmacological treasures can be present in only superficially examined ecosystems of our planet [23]. It is interesting to note here that the phylogenetic conservatism of RAS proteins had a very serious consequence: the large similarity of the bat and human ACE2 proteins made it possible for the COVID-19 virus, which used this particular protein as a cell surface anchor, to be transmitted from bats to humans and to adapt to the human molecule within only a few mutations (“variants of concern”), inducing a pandemic which recently killed millions of human beings [24]. 

## 4. Minor Variabilities of the Human RAS Genes

Minor variabilities of the human RAS genes can be important factors in hypertension and cardiovascular disease development. The time is approaching when—similar to present-day cancer molecular diagnostics—patient DNA analysis will contribute to the examination of pathomechanisms and the determination of individual optimal therapy. From the point of view of human biology, it would be very interesting to reveal which molecular alterations have accumulated under which geographical and ethnographic conditions. Two polymorphisms of the human ACE gene (*ACE I/D* and *ACE2 rs2106809*) have been reported which increase Ang II levels and elevate blood pressure, but at the same time have protective effects against severe malaria. Their frequency is elevated in malaria-endemic geographical areas [25]. The *ACE I/D* also increases the risk of hypertrophic cardiomyopathy [26], together with other variabilities of ACE [27]. Polymorphisms of the ACE and AT_1_R and AT_2_R genes seem to be associated with preeclampsia and pregnancy-induced hypertension [28]. Angiotensinogen is also a fairly conservative protein: several sequence features are maintained in 57 vertebrate species. In the human genome, however, minor variabilities are very frequent: 690 angiotensinogen variants have been identified in 1092 human genomes, the most frequent variations being somatic single nucleotide polymorphisms [29]. The potential pathological significance has yet to be investigated. 

## 5. The Diverse Functions of AT1R

The AT_1_R is distributed in diverse tissues, inducing diverse functions at the level of the organism. However, reflecting its long and complex development, this receptor has complex functions even at the molecular level. The pleiotropic effects of AT_1_R stimulation, almost unique among heterotrimeric G protein-coupled receptors, have been reviewed [1,2]. This receptor can associate, in addition to G_q/11_, G_i/o_ and G_12/13_ signal proteins. The termination of activation can be accomplished through the internalization of the receptor molecule via β-arrestin-mediated mechanisms. The substantial activation of the MAPK cascade reveals trophic effects and, interestingly, several dual-specificity MAPK phosphatases negatively regulating their activities are also upregulated in a negative feedback manner [3]. Earlier in the H295R human adrenocarcinoma cell line and in primary rat adrenocortical glomerular cells, we mapped Ang II-induced expression kinetics of *CYP11B2* and *BDNF* genes [30]. Later, in chronic in vivo studies, we found that the infusion of angiotensin II into rats resulted in reduced morphological lumen and the elevated thickness of the wall of resistance arteries, which only slowly and partially recovered after the cessation of the infusion [31]. In addition, aberrations in the geometry of the intramural coronary resistance artery network were observed [32]. A further complication is that the activation of AT_1_R by Ang II is accompanied by the release of endocannabinoids, as was found in a cell expression system [33] and in vascular tissues such as the skeletal muscle (gracilis) arteriole, coronary vessels or aorta [34,35]. The Ang II-stimulated release of 2-arachidonoylgylcerol was directly detected in aortic vascular smooth muscle cells [36]. In cardiac tissue, we also found that an Ang II-induced release of endocannabinoids may counteract the positive inotropic effect of Ang II to decrease metabolic demand [37]. Ang II also activates chronic inflammatory cellular pathways, inducing vascular damage and accelerating vascular aging (for a review, see [4]). In addition, according to some disputed views, ACE and Ang II even might restrict longevity throughout the animal world [38]. 

## 6. The Role of Ang II in Maintaining Blood Pressure under Basal Conditions

In terrestrial animals, salt and water conservation, blood volume conservation and blood pressure maintenance are inherently connected to each other. In the diverse functions of the renin–angiotensin–aldosterone system, this fact is reflected. The four most important actions of the renin–angiotensin system are all directed to elevate and maintain blood pressure: 1. Cellular (epithelial) sodium reuptake. 2. Stimulation of mineralocorticoid production (with similar effects). 3. Vascular smooth muscle contraction. 4. Inducing thirst. Among the physiological circuits controlling and maintaining normal blood pressure, those with the involvement of this octapeptide are among the most important. The renin gene and AT_1a_ receptor gene knock-out mice (homozygous) had blood pressure reductions of around 20–30 mmHg [39]. The RAS is active under basal conditions at “normal” levels of water and salt intake and fluid loss. Ang II is continuously produced in many tissues; the overdose of ACE inhibitor was found to decrease blood pressure to as low as 88/42 (57) mmHg (systolic pressure/diastolic pressure (mean pressure)) in a set of toxicology patients [40]. 

## 7. The Role of Ang II in Maintaining Blood Pressure under Extreme Conditions

The significance of having an effective renin–angiotensin system for human survival is even more prominent under extreme conditions. The mammalian brain (and to some degree heart) are extremely sensitive to any reduction in the continuous supply of oxygen through continuous blood flow. A drop in the mean arterial blood pressure below about 70 mmHg interferes with the cerebral blood flow and disturbs sensation, motor abilities, decision making and communication, and substantially reduces muscle force and physical and mental performance, in a real situation massively reducing the probability of survival, maybe even in a few minutes. Historically, our ancestors were subjected to an impressive array of dangerous blood pressure lowering effects, such as bleeding, crushing wounds, toxicoses, exsiccosis, water deprivation, excessive heat, infections, diarrhea, vomiting, profuse sweating, etc. Several blood pressure elevating mechanisms help to prevent this from occurring, and the renin–angiotensin system is one of the most powerful among them. In irreversible circulatory shock, plasma renin values enormously elevate, but even such extreme values cannot produce the blood pressure needed for adequate microcirculation. High plasma renin values can thus be considered a bad prognostic marker for critically ill patients [41].

## 8. Salt Appetite, Salt Preference and the Overconsumption of Salt

Behavioral elements are important in processes that control body water and salt. Ang II is a transmitter in hypothalamic, brain stem and limbic neuronal circuits controlling thirst, vasopressin release and salt preference. Salt preference increases when sodium deprivation is present. Several mammals have a salt appetite, and humans are among them. A salt appetite might have had an additional advantage in early humans turning hunters: even unsalted meat has a fairly salty taste [5,6].

Salt appetite and salt preference could have been important behavioral elements in keeping a salt (and fluid) balance in our early ancestors. We have good reason to think that salt preference, controlling salt intake, has been genetically adjusted to conditions when salt was scarce and the dangers of salt deficiency were larger than the dangers of consuming more salt than was optimal. However, with historical development, humans learned how to produce that commodity by evaporating sea water and by mining salt deposits from layers of solid rock. Hallstatt salt mining in the Austrian Alps dates back 7000 years, and in the bronze age in Europe there was substantial commercial salt transportation [42]. In Medieval times, the consumption of commercial salt turned habitual even among the poor, and salt taxation was one of the most stable entries in governmental income lists. In West and North European cities, foodstuffs preserved using salt became components of the everyday food of city dwellers, ensuring the development of high concentrations of non-agricultural urban residents with specific industrial, commercial and intellectual skills. Even lengthy deep-sea ship travels and military operations depended on salt-preserved food. With improved financial situations, limits on salt consumption practically disappeared and the genetically inherited salt taste dictating salt consumption started to elevate. This is an important element in the hypertension pandemic we are observing in the present day [43]. 

In developed countries, elevated salt consumption is—together with other factors—an important component of the increasing prevalence of the essential hypertension disease. Daily salt intake in practically all cultures now exceeds the estimated physiological requirements of 10–20 mmol/day [44]. The global sodium intake of adults runs at 4310 mg/day (10,780 mg/day salt), twice the recommended limit of the World Health Organization of 2000 mg/day (<5000 mg salt/day) [45] or the sodium limit advised by the US Institute of Medicine (2300 mg/day, 100 mmol/24 h) [46]. 

There is a classical negative feedback system between salt intake and the activity of the renin–angiotensin–aldosterone system, a system that works in the direction of salt accumulation [47]. In salt-sensitive individuals, anti-natriuretic effects are not sufficiently suppressed by salt consumption, which results in marked blood pressure elevation [48,49]. Reducing salt intake has a blood-pressure-lowering effect. According to the meta-analysis by He and McGregor [50], in the range of 3–12 g/day, the lower the salt intake, the lower the blood pressure. Salt restriction or the consumption of salt substitutes can be an alternative to drug treatment in certain hypertension cases [51]. 

## 9. The RAS as a Pharmacological Target in Large Populations

The enormous significance of these eight biologically inherited molecules of the RAS for human health can be judged from some statistics at hand. In the US, anti-hypertensive drug use increased from 20 to 27% of the population between 1999 and 2000 to 2011 and 2012, with 31% and 66% of the non-institutionalized population being involved in age groups 40–64 and >65 years, respectively. Of these, 12% took ACE inhibitors and 5.8% AT_1_R inhibitors [52]. In England, in 2018, 22% of primary care patients received antihypertensive prescriptions. ACE inhibitors being ordered increased from less than 0.5% in 1988 to over 9% in 2018, while angiotensin receptor blockers, which started to be produced in 1995, reached 4% in 2018 [53]. Among hypertensive treatments, in the years after 2011, ACE inhibitors represented 38% and angiotensin receptor blockers 2.1% [54]. Similarly high numbers, but with a different ratio between drug groups, have been reported from Denmark. Among 352 antihypertensive drug users per 1000 inhabitants, 70 were taking ACE inhibitors and 65 angiotensin receptor blockers [55]. Looking at such statistics, we can conclude that in a substantial part of the population of developed countries, especially in the elderly, we cannot talk any longer about the “physiological control of blood pressure and salt balance”: there is a combined pharmaco-physiological control, instead. 

## 10. Conclusions

We conclude that our RAS and salt preference behavior has not genetically adapted to the hedonistic salt use which is broadly accessible today. Public information should be more widely distributed about the risks of high salt consumption. Diagnostic tests and even genetic tests should be worked out to identify, within the supposedly heterogenous group of “essential hypertensives”, those for whom moderate or aggressive salt restriction can be an important element of therapy. Further, with such tests, a more targeted, individualized pharmacotherapy can be hopefully planned in the not-too-far future.

## Data Availability

Not applicable.
